# Multiple gene analyses of caligid copepods indicate that the reduction of a thoracic appendage in Pseudocaligus represents convergent evolution

**DOI:** 10.1186/1756-3305-6-336

**Published:** 2013-11-28

**Authors:** Mark A Freeman, Hilal Anshary, Kazuo Ogawa

**Affiliations:** 1 Institute of Ocean and Earth Sciences, University of Malaya, Kuala Lumpur, Malaysia; 2 Department of Fisheries, Laboratory of Fish Parasites and Diseases, Faculty of Marine Sciences and Fisheries, Hasanuddin University, Makassar, Indonesia; 3 Meguro Parasitological Museum, 4-1-1, Shimomeguro, Meguro-ku, Tokyo 153-0064, Japan

**Keywords:** *Pseudocaligus*, *Caligus*, Convergent evolution, Caligidae, Appendage reduction, *Lepeophtheirus*, Synapomorphy

## Abstract

**Background:**

The Caligidae is a family of parasitic copepods containing over 30 recognised genera. They are commercially important parasites as they cause disease in numerous finfish aquaculture facilities globally. Morphological features are used to distinguish between the genera and *Pseudocaligus* has traditionally been differentiated from *Caligus* solely by the presence of a much reduced form of the fourth thoracic leg. Currently there are numerous DNA sequences available for *Caligus* spp. but only the type species, *Pseudocaligus brevipedis*, has molecular data available, so systematic studies using molecular phylogenetic analyses have been limited.

**Methods:**

Three gene regions, SSU rDNA, 16S and CO1, for *Pseudocaligus fugu* from puffer fish from Japan and *Pseudocaligus uniartus* from rabbit fish from Indonesia are sequenced and molecular phylogenetic analyses performed in order to infer phylogenetic relationships between *Pseudocaligus* and other caligid copepods.

**Results:**

The analysis revealed that there was no discrete grouping of *Pseudocaligus* spp. and that they had a polyphyletic distribution within *Caligus* taxa. *Pseudocaligus fugu* grouped with *Caligus elongatus* and contained a unique synapomorphy in the SSU rDNA region only seen in members of that clade. *Pseudocaligus uniartus* formed a well-supported group, in the SSU rDNA analyses, with a *Caligus* sp. that also infects rabbit fish, but was unresolved in the other analyses. *Pseudocaligus brevipedis* consistently and robustly grouped with *Caligus curtus* and *C. centrodonti* in all analyses. The majority of *Lepeophtheirus* spp. form a monophyletic sister group to the *Caligus* clade; however, *L. natalensis* is unresolved in all analyses and does not form part of the main *Lepeophtheirus* clade.

**Conclusions:**

These findings do not support the morphological-based distinction between *Pseudocaligus* and *Caligus*, suggesting that the reduced fourth leg is a feature that has evolved on multiple occasions throughout caligid evolution. Congruent molecular phylogenetic data support groupings based on the presence of morphological features, such as lunules, geography and host fish type rather than appendage morphology. Therefore, we support the synonymy of *Pseudocaligus* with *Caligus*.

## Background

Parasitic copepods belonging to the family Caligidae are dorso-ventrally flattened ectoparasites that feed on the epithelium and blood of marine and brackish water fishes [1,2] but are also found on other marine animals such a whales [3]. They have the same general body plan consisting of a rounded cephalothoracic shield which is comprised of the head fused with four thoracic segments, a free fourth pedigerous segment, a genital complex and abdomen [1]. The presence or absence of features such as lunules and posterior sinuses, or the morphology of other features like the fourth pediger, genital complex, and the fourth leg are currently used to differentiate between the genera [2]. Some features such as the size of the fourth leg are extremely plastic within the group, ranging from totally absent (*Markevichus*), to vestigial (*Alebion*, *Pseudocaligus*, *Pseudolepeophtherius*) or well developed (most other genera) [4].

The Caligidae are globally important parasites that are collectively referred to as sea lice, and cause significant disease problems in marine aquaculture worldwide. They can affect fish health and growth rates due to their feeding activities, which causes skin lesions that lead to osmotic regulatory problems and eventually to mortalities [5]. In salmon farms, *Lepeophtheirus salmonis* infecting fish in Europe and *Caligus rogercrosseyi* infecting fish in Chile have caused significant losses with numerous treatments being required to effectively control their populations [6,7]. It has been estimated that the global annual cost attributed to sea lice infections, in salmonid aquaculture alone, was greater than 300 million Euros in 2006, which constitutes an average of 6% of the value of production for the countries affected by such infestations [8]. However, these estimates do not take into consideration the additional costs to the environment associated with the use of chemical parasiticides or welfare aspects concerning the fish, farm staff and ultimately the consumer.

Caligids from the genus *Pseudocaligus* have not been widely reported as being pathogenic to farmed fish. However, recently *P. fugu* has been reported to cause skin lesions in farmed tiger puffer, *Takifugu rubripes*, in Japan [9,10] and *P. uniartus* is causing serious disease outbreaks in the developing aquaculture of rabbit fish, *Siganus* spp. in the Philippines [11] and more recently Indonesia. *Pseudocaligus* spp. have been described as taxonomically distinct from other sea lice, such as *Caligus*, as they have a significantly reduced fourth leg, which is well developed in most other caligids. However, numerous researchers have questioned the validity of *Pseudocaligus* as a genus [1,12,13] and a recent review on caligid systematics, based on morphological features, concluded that *Pseudocaligus* should be treated as a junior synonym of *Caligus*[14], with the nomenclatural revisions arising from that decision clarified by Özak *et al.*[15]. However, throughout this manuscript we refer to members of the recently synonymised genus *Pseudocaligus* for reasons of clarity.

Currently, there is a paucity of DNA sequence data available for pseudocaligid taxa*,* with the majority of molecular phylogenetic studies of caligids focusing on the genera *Caligus* and *Lepeophtheirus*[12,16]. In the present study, we aim to provide additional DNA sequences for *P. fugu* and *P. uniartus* and include these in molecular phylogenetic studies of the Caligidae.

## Methods

*Pseudocaligus uniartus* were collected from naturally infected rabbit fish, *Siganus guttatus*, reared in the Marine Research Station located in Barru Regency, South Sulawesi and *P. fugu* were obtained from naturally infected tiger puffer, *Takifugu rubripes* in Nagasaki Public Corporation, Japan. DNA samples were analysed from 4 individuals of *P. uniartus* and 3 individuals from *P. fugu*, which had been preserved in 70% ethanol. The DNA was extracted from whole copepods using QIAamp DNA Mini Kit (Qiagen Inc., Hilden, Germany) following the manufacturer’s tissue protocol. DNA was eluted in water and stored frozen at −20°C prior to PCR amplification.

### PCR and sequencing

The target regions for PCR amplification were the small subunit ribosomal DNA (SSU rDNA) and the mitochondria genes CO1 and 16S ribosomal RNA gene. The oligonucleotide primers used to amplify the target regions were: SSU rDNA universal primers 390fwd (5-AGAGGGAGCCTGAGAAACG-3), 870rev (5-GTTGAGTCAAATTAAGCCGCA-3), 870fwd (5-TGCGGCTTAATTTGACTCAAC-3) and 18gM (5-CTTCCGCTGGTTC-ACCTACG-3) [10,17] to get nucleotide sequences of around 1300 bp. In addition, to obtain more complete sequence reads, a specific reverse primer was designed (Pcalrev: 5-CCTCCAATTGTTCCTCGTT-3) in conjunction with the forward 18e universal primer (5 CTGGTTGATTCTGCCAGT 3) [18]; 16S primers 16ceoioiF (5-GCCTGTTTATCAAAGACATA-3) and 16ceoioiR (5-ATAGAAACCAATCTGGCTTA-3) [12]; CO1 primers CO1fwd (5-AGWGGRTTTTGATCHGGNYT-3) and CO1rev (5-GGRTCAAAAAAYSTDGTRTTTA-3) [16].

All PCRs were performed in 20 μL volumes containing dNTPs 0.2 mM, primers 0.8 μM, Taq polymerase 0.02 U/μL, 2 μL 10× buffer PCR and milli-Q water to achieve the correct final volume. PCR conditions for 16S was as follows: denatured at 98°C for 2 min, followed with 40 cycles (95°C for 30 s, 46°C for 30 s, 72°C for 1 min) and final elongation 72°C for 5 min. For CO1: initial denaturation at 98°C for 2 min, followed with 37 cycles (95°C for 30 s, 50°C for 30 s, 72°C for 1 min) and final extension at 72°C for 2 min. For SSU rDNA: initial denaturation at 98°C 5 min, followed with 35 cycles (95°C for 30 s, 55°C for 45 s, 72°C for 1 min), and final extension at 72°C 7 min. PCR amplifications were performed in an iCycler (Bio-Rad, Hercules, CA, USA) thermocycler and the products visualised in an SYBR green stained 1.5% agarose gel using a 100 bp ladder (Takara) to estimate the size of the amplicons.

PCR products were purified using PCR purification or agarose gel extraction kits (Qiagen), depending on whether bands were excised from gels to reduce contamination, and used directly in sequencing reactions at the Operon Biotechnologies Company, Tokyo, Japan. Both forward and reverse directions were sequenced for all products using the same primers from the initial amplifications. DNA sequencing was performed on all positive PCR products of the expected sizes for 4 individual replicates for *P. uniartus* and 3 replicates for *P. fugu*. Nucleotide Basic Local Alignment Search Tool (BLAST) searches were performed for each sequence to confirm a copepod origin.

### Sequence alignment and phylogenetic analyses

The sequences were analyzed using Edit Sequence of DNASTAR Lasergene version 7.2.1 and Bioedit Sequence Alignment Editor 7.0.5.3 comparing with the original chromatogram. Multiple Sequence Alignment (MSA) with all reported caligids in the GenBank was performed using software Clustal X and then edited using Bioedit.

Phylogenetic analyses were performed using the maximum likelihood methodology in PhyML [19] with the general time-reversible substitution model selected and 1000 bootstrap repeats, and Bayesian inference (BI) analysis using MrBayes v. 3.2 [20]. For the BI analysis, models of nucleotide substitution were first evaluated for the alignment using MrModeltest v. 2.2 [21]. The optimum evolutionary model based on the Akaike information criterion (AIC) was the general time-reversible GTR + I + G model of evolution where G is the gamma distributed rate variation among sites and I is the proportion of invariable sites. Therefore, the settings used for the analysis were nst = 6, with the gamma-distributed rate variation across sites and a proportion of invariable sites (rates = invgamma). The priors on state frequency were left at the default setting (Prset statefreqpr = dirichlet (1, 1, 1, 1)). Posterior probability distributions were generated using the Markov Chain Monte Carlo (MCMC) method with four chains being run simultaneously for 1000,000 generations. Burn in was set at 2500 and trees were sampled every 100 generations making a total of 7500 trees used to compile the majority rule consensus trees.

## Results

### PCR Amplification and DNA sequencing

PCR amplification and DNA sequencing for the three gene regions for both *Pseudocaligus* was successful. BLAST searches of SSU rDNA sequences of 1772 bp for *P. fugu* and 1776 bp for *P. uniartus* showed high identities to *Caligus elongatus* (99%) and an unidentified *Caligus* sp. (98%) from rabbit fish respectively. BLAST searches of 16S mitochondrial sequences of 442 bp for *P. fugu* and 457 bp *P. uniartus* showed a much lower identity of 81% to *Caligus gurnardi* and 82% to *Caligus centrodonti* respectively and BLAST searches of CO1 mitochondrial sequences of 567 bp for *P. fugu* and 566 bp *P. uniartus* revealed an 81% identity to isolates of *C. elongatus* and an 83% identity to *Caligus clemensi* and *C. centrodonti* respectively. DNA sequences obtained in this study were submitted under the accession numbers [GenBank: KC569363-KC569368].

### Phylogenetic analysis of Pseudocaligus within the Caligidae

**Figure 1:**
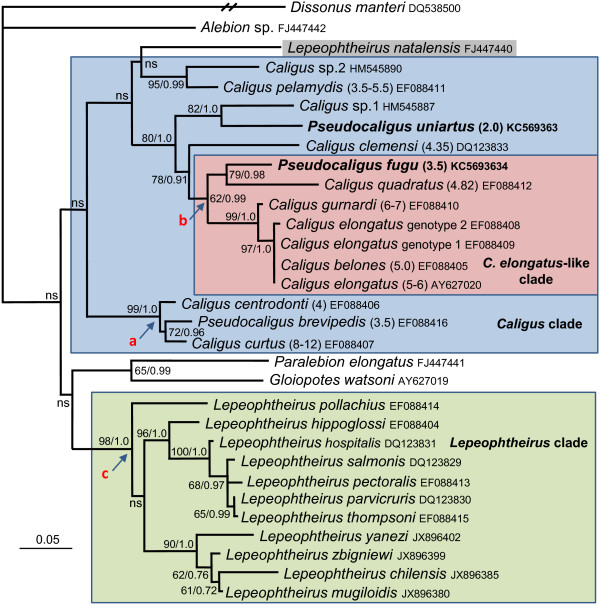
**Analyses of SSU rDNA of 31 caligid taxa.** Maximum likelihood (ML) phylogeny of the Caligidae based on 1685 characters of aligned SSU rDNA sequence data. The three *Pseudocaligus* spp. are placed in the *Caligus* clade (blue box), although support for the overall grouping is low. *Pseudocaligus brevipedis* is robustly supported (node a) in a clade with *C. centrodonti* and *C. curtus* that form a basal clade in the *Caligus* group. *Pseudocaligus fugu* is moderately well supported, from node b, as a member of the *Caligus elongatus*-like clade (red box) within the larger *Caligus* grouping. *Pseudocaligus uniartus* is well supported forming a sister clade with *Caligus* sp. 1 to the *C. elongatus*-like clade. All but one *Lepeophtheirus* spp. form a very robust clade, *Lepeophtheirus* clade (green box) from node c, which forms a consistent but poorly supported sister clade to the *Caligus* group (blue box). However, *L. natalensis* (highlighted) does not group with other *Lepeophtheirus* spp. and forms an unsupported branch at the base of the *Caligus* clade. Numbers in parentheses after specific names in the *Caligus* clade denote recorded sizes of adult female copepods from the literature [1,22-24]. Numbers at the nodes represent branching support using non-parametric bootstraping (ML 1000 replications) and Bayesian posterior probabilities. Nodes with a bootstrap support of <50 and Bayesian posterior probability <0.95 were considered not supported (ns). *Dissonus manteri* is used as an outgroup and to root the tree.

**Figure 2:**
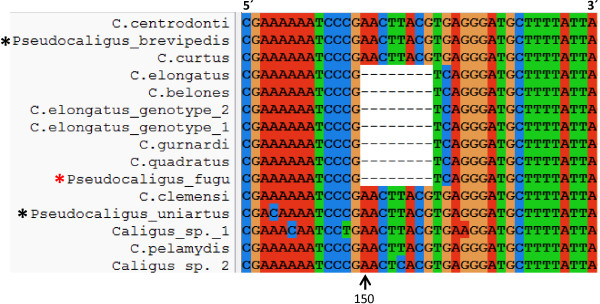
**Synapomorphy in the SSU rDNA region of members of the *C. elongatus*-like clade.** Part of an alignment of 18S Caligidae sequences showing an eight base pair deletion starting 150 bases from the 5′ end of our alignment, immediately after the motif sequence AAAAAATCCCG. This deletion is only seen in the seven members of the *Caligus elongatus*-like clade (red box Figure 1), which includes *P. fugu* (red asterisk), but not other *Pseudocaligus* spp. (black asterisk).

**Figure 3:**
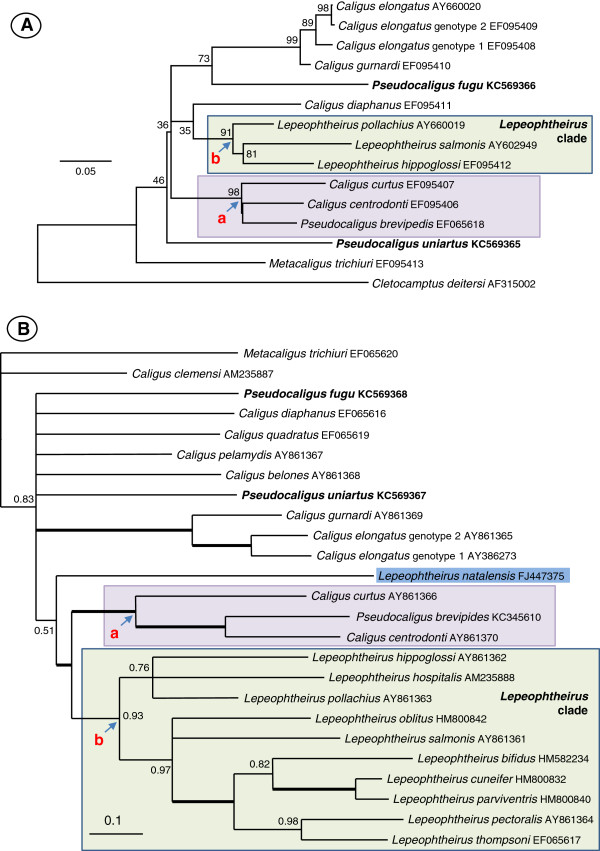
**Phylogenetic trees of mitochondrial DNA sequence analyses for the Caligidae. A)** Maximum likelihood tree constructed from analysis of 462 characters of aligned small subunit 16S ribosomal gene. **B)** Bayesian topology based on analysis of 528 characters of aligned CO1 genes of 25 caligid taxa. *Pseudocaligus brevipedes* is robustly supported in a clade with *C. curtus* and *C. centrodonti* in both analyses (node a, shaded boxes). *Pseudocaligus fugu* forms the basal taxon to the *C. elongatus* group in the 16S analysis and is unresolved in the CO1 tree. *Pseudocaligus uniartus* is unresolved in both analyses. *Lepeophtheirus* spp. form well-supported clades in both trees (node b, green boxes), apart from *L. natalensis* (highlighted) in the CO1 tree that is unresolved. Thick branches are from nodes with full support and figures at the nodes represent bootstrap support values from 1000 samplings for the 16S tree and posterior probabilities for the Bayesian topology.

The DNA sequences for *Pseudocaligus* did not group together to form a discrete monophyletic clade in any of our phylogenetic analyses. *Pseudocaligus fugu* was placed in a clade with *C. elongatus* in two analyses, being robustly supported in SSU rDNA analyses within the *C. elongatus*-like clade (Figures 1 and 2), well supported in the 16S analysis but unresolved along with many *Caligus* spp. in the CO1 analysis (Figure 3A and B). *Pseudocaligus brevipedis* was robustly supported in a clade containing *C. curtus* and *C. centrodonti* in all three analyses (Figures 1 and 3), and formed a sister clade to a well supported *Lepeophtheiru*s clade in the CO1 analysis (Figure 3B). *Pseudocaligus uniartus* was well supported in the *Caligus* clade in the SSU rDNA phylogenies but remained unresolved in all other analyses (Figure 1, Figure 3A and B).

An alignment of 31 caligid SSU rDNA sequences produced congruent topologies using maximum likelihood and Bayesian methodologies (Figure 1). The three *Pseudocaligus* spp. are robustly placed in the *Caligus* clade, although support for the overall grouping and the basal branching events in the tree are low (Figure 1). *Pseudocaligus fugu* is well supported as a member of the *C. elongatus*-like clade within the larger *Caligus* grouping. *Pseudocaligus uniartus* is well supported and forms a sister clade with *Caligus* sp. 1 to the *C. elongatus*-like clade. *Pseudocaligus brevipedis* is robustly supported in a clade with *C. centrodonti* and *C. curtus* that form the basal clade in the *Caligus* group. The majority of *Lepeophtheirus* spp. form a robust clade, which is a sister group to one containing other non-lunule bearing caligid genera, *Paralebion* and *Gloiopotes*, together forming a sister clade to the *Caligus* group. However, *L. natalensis* does not group within the main *Lepeophtheirus* clade and forms an unsupported branch at the base of the *Caligus* clade (Figure 1). During the alignment of 18S sequence data an eight base pair deletion starting immediately after the motif sequence 5′AAAAAATCCCG was found to occur (Figure 2), but was only present in the seven members of the *Caligus elongatus*-like clade, which includes *P. fugu*, but not the other *Pseudocaligus* spp. (Figure 1).

## Discussion

The family of parasitic copepods, the Caligidae, comprises some 33 accepted genera [25], of which only 7 have DNA sequence data available that can be used to infer molecular phylogenetic relationships. It is not possible to provide a comprehensive molecular phylogeny for the family until more DNA data for the various genera become available. However, available molecular data largely come from two of the most specious genera, *Caligus* and *Lepeophtheirus*, and can be used to identify important molecular phylogenetic relationships between these related taxa. Morphologically, *Lepeophtheirus* differ from *Caligus* as they lack lunules. Lunules are paired sucker-like structures on the frontal plates that are used for attachment to the fish host, and are unique to nearly half of the genera within the Caligidae, including *Caligus* and *Pseudocaligus*[2,26]. Lunules are thought to have evolved only once in the Caligidae and character based phylogenetic analysis of the family suggests that several genera, such as *Lepeophtheirus*, have secondarily lost their lunules [2]. In accordance with this theory, we consistently retrieve the majority of *Lepeophtheirus* spp. as a separate well-supported clade in our analyses, with the lunule bearing *Caligus* and *Pseudocaligus*, grouping together as a sister taxon. However, within the lunule-bearing group, the three sequences available for *Pseudocaligus* are consistently placed apart from each other, and form parts of recoverable clades, that appear to reflect the geographical location and/or the fish host taxon. In the SSU rDNA analyses, *P. fugu* groups with *Caligus quadratus* also infecting fish from Japan and forms part of the *C. elongatus* clade; *P. uniartus* groups with an unidentified *Caligus* sp. that also infects siganid fish from Indo-Pacific region, and *P. brevipedis*, found on gadoid fish in the Atlantic, consistently groups with two species of *Caligus* from the Atlantic, one is the type species of the genus, *C. curtus*, which is also found on gadoid fish. Interestingly, this robustly places the type species for *Pseudocaligus*, *P. brevipedis*, together with the type species for *Caligus*, but suggests that the lineages strongly reflect zoogeographic history and host fish taxon rather than morphology. It is also interesting, in the SSU rDNA and CO1 analyses that *Lepophtheirus natalensis*, the only *Lepeophtheirus* sequence known solely from an elasmobranch, groups away from an otherwise well supported clade containing all *Lepeophtheirus* sequences from teleosts (Figures 1 and 3B) and does not group with other shark-infecting caligid species, such as *Paralebion elongatus* (Figure 1). This is supported by greater genetic distances (data not shown) between *L. natalensis* and other *Lepeophtheirus* sequences compared to other caligid genera and suggests that when more DNA data is available for the genus, that *Lepeophtheirus* as currently constituted and identified will not form a monophyletic group. In this study we analysed one nuclear (SSU rDNA) and two mitochondrial genes (CO1 and 16S) and found a reproducible level of congruence for some caligid taxa between the phylogenies. However, numerous nodes remain unsupported in all trees that would otherwise allow a far better inference of phylogenetic relatedness between the species and genera within the Caligidae. Whilst the inclusion of more caligid taxa will undoubtedly lead to a better resolution of phylogenetic relationships within the family, it may also be useful to seek additional gene regions that are less conserved than the SSU rDNA but not as variable as the mitochondrial genes. The 5.8S region of the ribosomal RNA gene and the flanking internal transcribed spacer regions (ITS1 and ITS2) have been used successfully for some phylogenetic analyses, however, the ITS regions are known to be highly divergent among crustaceans and may only be an appropriate marker for studies at the species or population level [27]. Potentially, utilising a concatenated set of conserved gene regions from nuclear rDNA may provide sufficient information for good resolution at the generic level for molecular systematic studies of the Caligidae.

There were only nine species of *Pseudocaligus* recognised (Table 1), which is considerably smaller than the number of *Caligus* species described, currently in excess of 240 [25]. Of these nine, the majority are found in the Indo-Pacific region except for the type species *P. brevipedis* and *P. apodus* that are found in European waters (Table 1). It is interesting that four of the seven species of *Pseudocaligus* from the Indo-Pacific region are found infecting puffer fish, Tetraodonitidae (Table 1). This either demonstrates an unambiguous correlation between host fish family and the evolutionary reduction of the fourth leg, or implies that the *Pseudocaligus* infecting puffer fish in the Indo-Pacific region have radiated from a common ancestor with a *Pseudocaligus*-like morphology. If the latter is the case, then we would expect *Pseudocaligus* from puffer fish from the Indo-Pacific to group with *P. fugu* in future molecular phylogenetic studies and to have the same synapomorphy seen in the SSU rDNA sequence (Figure 2). It is also noteworthy that there appears to be a trend toward miniaturisation in pseudocaligids (Table 1), *Pseudocaligus uniartus*, from the present study, being the most extreme example at only 2 mm in length for an adult female. However, if adult female size data is considered with respect to SSU rDNA phylogenetic placements for the type species, *C. curtus* and *P. brevipedis*, the opposite trend can be observed (Figure 1). *Caligus curtus* and *P. brevipedis* form a well-supported clade from node a (Figure 1), with *C. centrodonti* as the basal taxon measuring about 4 mm [1]. The derived taxa measure a comparable 3.5 mm for *P. brevipedis*[28], whilst *C. curtus* is significantly larger with a range of 8–12 mm [24]. A similar trend can be seen with *P. fugu* (from node b, Figure 1) where the derived taxa *C. elongatus*, *C. belones* and *C. gurnardi* are all larger in size [1,24]. This suggests a relationship between body length and the presence of a functional fourth leg, where in our SSU rDNA analyses the evolutionary derived species are larger and possess fully developed fourth legs. However, in the two mitochondrial gene phylogenies, the larger *C. curtus* is well supported as basal to the smaller *C. centrodonti* and *P. brevipedis*. It is apparent that the size of the fourth leg is extremely plastic within the Caligidae, and appears likely that fourth leg reduction or retention occurs readily during evolution and may be a function of adaptation to host fish or new environments, and may also be linked to body length. Additional DNA data from related caligids will be required to clarify these phylogenetic inconsistencies.

**Table 1:** ***Pseudocaligus *** **spp. (recently synonymised with *Caligus *** **[**[14]**,**[15]**]), with fish host, geographical location and sizes**

***Pseudocaligus *** **spp.**	**Host fish in**	**Geographical location**	**Size female (mm)**	**Size male (mm)**
*P. apodus* Brian, 1924 [29]	*Mugil* sp. (Mugilidae) and *Eugaleus galeus* (Triakidae)	Mauritania	5-6	n/a
*P. brevipedis* Bassett-Smith, 1896 (type) [1,28]	*Motella tricirrata*	UK	3.5	< female
	(Lotidae, Gadiformes)			
*P. fistulariae* Pillai, 1961 [30]	*Fistularia petimba*	India (Arabian Sea)	5.1	3.2
	(Fistulariidae)			
*P. fugu* Yamaguti, 1936 [31]	*Takifugu* spp.	Japan	3.3-3.63	
	(Tetraodonitidae)			
*P. indicus* Hameed, 1977 [32]	*Dactyloptena orientalis*	India (Arabian Sea)	4.5	3.9
	(Dactylopteridae)			
*P. laminatus* Rangnekar, 1955 [33]	*Tetrodon oblongus*	India (Arabian Sea)	3.64	1.55
	(Tetraodonitidae)			
*P. parvus* Bassett-Smith, 1898 [34]	*Tetrodon oblongus*	India (Arabian Sea)	3.4	2.3
	(Tetraodonitidae)			
*P. subparvus* Hameed, 1977 [32]	*Arothron hispidus*	India (Arabian Sea)	3.5	n/a
	(Tetraodonitidae)			
*P. uniartus* Ho, Kim, Cruz-Lacierda et Nagasawa, 2004 [11]	*Siganus guttatus*	Philippines	2.0	1.9

The phylogenetic distribution of the three sequenced gene regions now available for pseudocaligids indicates that the genus is polyphyletic, i.e. these species do not have a common ancestor with a reduced fourth leg. We infer that this trait evolved independently for each of the three *Pseudocaligus* species sequenced so far, suggesting that they have all been subjected to similar evolutionary pressures. Kabata [4] suggested that the fourth leg has become largely, if not entirely, non-functional, and plays no discernible role in locomotory activities, and therefore it would atrophy. Indeed, similar traits are also observed in other caligid genera; *Pseudolepeophtheirus* contains a copepod with vestigial fourth leg appendages and in the genus *Markevichus* they have been completely lost. The development of the fourth leg is similar in *Caligus* and *Lepeophtheirus*, first appearing as rudimentary and lobate at the first chalimus and developing to a ventrolaterally paired anlagen of the fourth leg by the second chalimus and being a fully developed uniramus appendage prior to the final molt to adult [13]. However, in *Pseudocaligus*, the fourth leg first appears at the second chalimus and is suppressed from then, never fully developing [9]. Although reduction or loss of the fourth leg is neither an isolated nor a phylogenetically related occurrence, it is still only apparent in the minority of described caligids. Retention of the fourth leg in the majority of the Caligidae suggests that they are still functional and may be important for ease of movement on fish hosts in larger caligids.

Arthropods exhibit remarkable body plan diversity that includes variation in the number, shape, and size of limbs, with crustaceans having a higher degree of appendage specialization than any other animal group [35]. Several studies have demonstrated that evolutionary changes in arthropod body plan are linked to changes in the expression of Hox genes [36] including the Ultrabithorax (*Ubx*) gene in the arrangement of distinct thoracic appendage identities in crustaceans [37]. Therefore, it is plausible that during caligid evolution, changes in the Hox gene have resulted in a reduction of thoracic limbs to occur in parallel in different populations of parasitic copepods.

In their molecular phylogenetic studies of the Caligidae, Øines and Shram [12] also found that *P. brevipedis* was closely associated with *Caligus* spp. in contrast to the character-based study of Ho and Lin [2], where *Pseudocaligus* was estimated to be more distant to the genus *Caligus*. They proposed on the basis of their molecular phylogenetic data that *P. brevipedis* should be included into the genus *Caligus* based on its close association with *C. curtus*, *C. centrodonti*, and other *Caligus* species. Other researchers, on the basis of morphology and developmental features, also expressed doubts about the validity of *Pseudocaligus*[1,13]. Kabata [1] believed that both the genera *Pseudolepeophtheirus* and *Pseudocaligus* should be synonymized with their parent genera, and highlighted the fact that another caligid genus, *Pseudoanuretes*, contains taxa with both normal and vestigial fourth legs, indicating that reduction was not an anomalous trait within the Caligidae. Our data confirms and supports this consensus of opinion, that *Pseudocaligus* is not a valid genus in the Caligidae.

## Conclusions

We have demonstrated, with molecular phylogenetic studies of caligid copepods, that pseudocaligids group with other lunule bearing copepods from the genus *Caligus*, but they have a polyphyletic distribution within the group, and tend to group with geographically related *Caligus* spp. or with those infecting related host fish. We infer that the reduction of the fourth leg has evolved independently on multiple occasions in the Caligidae and is an example of convergent or parallel evolution within a related group of organisms. Therefore, we support the synonymy of *Pseudocaligus* with *Caligus*.

## Competing interests

The authors declare that they have no competing interests.

## Authors’ contributions

HA and MAF carried out the molecular part of the study and phylogenetic analyses. KO collated the publication history and taxonomic information. MAF, HA and KO all helped to prepare the manuscript and were all agreed upon the final version.
